# Nutritional Characterization of *Prosopis laevigata* Legume Tree (Mesquite) Seed Flour and the Effect of Extrusion Cooking on its Bioactive Components

**DOI:** 10.3390/foods7080124

**Published:** 2018-08-01

**Authors:** Luis Díaz-Batalla, Juan P. Hernández-Uribe, Roberto Gutiérrez-Dorado, Alejandro Téllez-Jurado, Javier Castro-Rosas, Rogelio Pérez-Cadena, Carlos A. Gómez-Aldapa

**Affiliations:** 1 Área Académica de Química, Universidad Autónoma del Estado de Hidalgo, Carretera Pachuca-Tulancingo Km 4.5, Mineral de la Reforma, C.P. 42184 Hidalgo, Mexico; ldiaz@upfim.edu.mx (L.D.-B.); jcastro@uaeh.edu.mx (J.C.-R.); 2 Ingeniería Agroindustrial, Universidad Politécnica de Francisco I. Madero, Tepatepec, C.P. 42660 Hidalgo, Mexico; 3 Instituto de Ciencias Agropecuarias, Universidad Autónoma del Estado de Hidalgo, Av. Universidad Km 1, Rancho Universitario, Tulancingo de Bravo, C.P. 43600 Hidalgo, Mexico; hdezspark@hotmail.com; 4 Facultad de Ciencias Químico Biológicas, Universidad Autónoma de Sinaloa, Ciudad Universitaria, Culiacan, C.P. 80040 Sinaloa, Mexico; robe399@hotmail.com; 5 Departamento de Biotecnología, Universidad Politécnica de Pachuca, Zempoala, C.P. 43830 Hidalgo, Mexico; alito@upp.edu.mx (A.T.-J.); rpcadena1904@gmail.com (R.P.-C.)

**Keywords:** mesquite, *Prosopis laevigata*, extrusion, phenolic compounds, radical scavenging capacity, apigenin

## Abstract

Mesquite (*Prosopis laevigata*) is a legume tree widely distributed in Aridoamerica. The mature fruit of this legume is a pod, which is currently underutilized and has high nutritional potential. In the present work, mesquite seed flour is described in terms of its nutritional value, as well as the effect of extrusion cooking on its bioactive components. Mesquite seed flour is rich in fiber (7.73 g/100 g) and protein (36.51 g/100 g), with valine as the only limiting amino acid. Total phenolic compound contents in raw and extruded seed flour were 6.68 and 6.46 mg of gallic acid equivalents/g (mg GAE/g), respectively. 2-2-Diphenyl-1-picrylhydrazyl (DPPH) radical scavenging capacity values in raw and extruded seed flour were 9.11 and 9.32 mg of ascorbic acid equivalent/g (mg AAE/g), respectively. The absorbance at 290 nm, as an indicator of generation of Maillard reaction product (MRP), was the same for raw and extruded samples. Apigenin was the only flavonoid found in mesquite seed flour (41.6 mg/kg) and was stable in the extrusion process. The water absorption index (WAI) and water solubility index (WSI) were changed significantly during extrusion. The expansion of mesquite seed flour extrudates was null due to the high protein and fiber content in the sample. Extrusion cooking of mesquite seed flour is a useful form of technology for the industrialization of this underutilized and nutritionally valuable legume.

## 1. Introduction

Legumes have been an essential part of the human diet for centuries, with a major role in global food security, environmental challenges, and healthy diets [1]. Legumes have mastered symbiotic nitrogen fixation, leading to significant advantages for agricultural sustainability; however, they contribute to just a small portion of staple foods worldwide compared with cereals [2]. A shift in land use towards grain legumes would substantially lower the carbon footprint of the production of protein for human consumption [2]. Consumption of legumes seeds contributes to reduced risk of mortality because of their benefits against major chronic diseases and their risk factors due to their bioactive components [3,4]. Orphan crops are minor crops with regional importance that have been largely neglected by researchers and industry due to limited economic importance in the global market [2]. Orphan food legumes are usually grown in arid regions, often on marginal land unsuitable for major crop species. They have heat- and drought-tolerant traits and high nutritional value [2,4]. Wild and underutilized legumes with high nutraceutical value should be explored for overcoming protein energy malnutrition. The presence of anti-nutrients in legume seeds might not be an impediment, as proper processing methods can make them edible for a safe use [5]. Extrusion cooking technology is a high-temperature, short, and versatile food operation that converts agricultural raw materials into fully cooked and shelf-stable food products with enhanced nutritional attributes [6]. Legume extrusion cooking eliminates anti-nutritional factors and improves protein digestibility at a cost lower than other cooking systems [6,7]. Legumes could be included as protein sources in the extrusion process to formulate nutritionally enhanced functional food products [6].

The genus *Prosopis* is comprised of a group of nitrogen-fixing trees belonging to the Fabaceae family distributed in arid and semiarid regions of Asia, Africa, and America. *Prosopis* species were a major staple food for indigenous peoples in arid regions of America before the arrival of Europeans [8]. The mature fruit of the genus *Prosopis* is an indehiscent pod formed of an exocarp, a developed mesocarp, and a woody endocarp which protects the seed [8]. Pod flour of *Prosopis* is a versatile ingredient with high potential for the food industry. It is rich in protein, sugars, and fiber, and is gluten-free [8,9,10]. Pods of the *Prosopis* species have been reported as a source of bioactive compounds with antioxidant, inflammatory, and antihypertensive activities [8,11,12,13]. Seed flour of *Prosopis alba* showed high levels of proteins, minerals, fiber, and phenolic compounds, mainly flavones, with low content of total carbohydrates and fats [9,14]. Pod flour of *Prosopis laevigata* is a good source of lysine, sulfur-containing amino acids, and total phenolic compounds, with higher radical scavenging capacity than soybeans and common beans [15]. Thermal treatment of *P. laevigata* flours (for example in a baking process) increases the apparent total phenolic compound content and radical scavenging capacity, an effect associated with the generation of Maillard reaction products (MRPs) [15]. In order to increase the description of the nutritional value of the *Prosopis* genera, and explore technological options for its processing, in the present work the seed flour of the specie *P. laevigata,* widely distributed in Aridoamerica, is described in its nutritional value, and the bioactive compounds content is analyzed in raw and extruded mesquite seed flour. 

## 2. Materials and Methods

### 2.1. Chemicals 

Gallic acid, 2-2-diphenyl-1-picrylhydrazyl (DPPH), Folin–Ciocalteu’s phenol reagent, daidzein, genistein, myricetin, quercetina, kaempferol, and apigenin were from Sigma (St. Louis, MO, USA).

### 2.2. Plant Material and Preparation of Mesquite Seed Flour 

Dry mature pods were collected from trees of *P. laevigata* at the experimental field of the Universidad Politécnica de Francisco I. Madero in the semiarid region of the Mezquital Valley in Hidalgo, Mexico. Collected yellow-brown mature pods were ground using a 900-W blender (Nutribullet, Los Angeles, CA, USA). Milled pods was sieved in a 30 mesh, retaining the intact endocarp. The intact endocarp was submitted to a second milling process, producing seed flour which passed through an 80 mesh and the retained brans.

### 2.3. Seed Flour Chemical Composition 

Crude fat, protein, moisture, fiber, and ash contents of seed flour were determined according to the procedures described in the Association of Official Analytical Chemists (AOAC), methods 920.39, 992.15, 925.09, 991.43, and 923.03, respectively [16].

### 2.4. Seed Flour Amino Acid Profile

The amino acid content in raw flour was analyzed after hydrolysis with 6 N HCl, using a cation exchange separation column (LCA K06/Na, 4.6 × 150 mm; Sykam GmbH, Eresing, Germany) with ninhydrin postcolumn derivatization, in an amino acid analyzer (Sykam GmbH, Eresing, Germany) [15]. The same method was used for sulfur-containing amino acids, using performic acid oxidation before hydrolysis [17]. Tryptophan was determined at 620 nm, after enzymatic hydrolysis with papain, and reaction with p-dimethylaminobenzaldehyde [18].

### 2.5. Seed Flour Extrusion

Seed flour was conditioned with purified water to obtain a moisture content of 16%. Seed flour was extruded in single screw extruder (Brabender 19/25DN, Duisburg, Germany) equipped with a 19-mm diameter and 3:1 compression ratio screw, working at 170 rpm. Barrel temperatures were 80, 100, 120, and 150 °C for zones 1, 2, 3, and 4, respectively. Feeder rate was set at 30 rpm and the diameter of the exit die was 3 mm. Seed flour extrudate was dried and ground until it passed through 80 mesh. 

### 2.6. Preparation of Extracts

Raw seed flour and extruded seed flour were extracted with aqueous ethanol [15]. Samples of 100 mg were extracted with one milliliter of 40% ethanol in water (*v/v*) and centrifuged at 12,000 rpm/10 min. The extract was removed and the extraction process was done again with the residual pellet. Both extracts were mixed and diluted with 40% ethanol to obtain a final volume of 25 mL. These extracts were used for total phenolic compound content, radical scavenging capacity, and absorbance at 290 nm. For flavonoids, a replicate was prepared for each obtained extract and they were submitted to HCl hydrolysis with ethyl acetate aglycone recovery [19]. The ethyl acetate was evaporated at 45 °C and the residue was diluted in absolute ethanol to obtain a final volume of 2 mL.

### 2.7. Total Phenolic Compounds

Total phenolic compounds content was determined in raw and extruded seed flour extracts by the Folin–Ciocalteu reagent method [11]. The absorbance was measured at 760 nm and the results were expressed as mg of gallic acid equivalents/g (mg GAE/g) of dry weight.

### 2.8. DPPH Radical Scavenging Capacity 

The radical scavenging capacity of raw and extruded seed flour extracts was determined using the DPPH synthetic radical method [11]. The absorbance was measured at 515 nm and the results were expressed as mg of ascorbic acid equivalent/g (mg AAE/g) of dry weight.

### 2.9. Ultraviolet Analysis of Maillard Reaction Products (MRPs) 

Analysis of MRPs in extracts of raw and extruded flours was performed using the spectrophotometric method reported by Yu et al. [20]. Appropriate dilutions of extracts were scanned from 240 to 320 nm using an ultraviolet-visible (UV-VIS) spectrophotometer (Genesys 10 S, Thermo Scientific, Waltham, MA, USA). The presence of MRP was evidenced by the increase in the UV absorbance at 290 nm.

### 2.10. Flavonoids 

The flavonoid content was determined by reversed-phase high-performance liquid chromatography (RP-HPLC) using a Dionex Ultimate 3000-DAD system (Thermo Scientific) supplied with an Acclaim 120 C-18 (4.6 × 100 mm) column [21]. For separation, solvent A was water adjusted with acetic acid to pH 2.8, and solvent B was acetonitrile. For flavonoid elution, the gradient was linear to 30% B in 5 min, 45% B in 8 min, and 55% B in 14 min; afterwards the column was washed with 95% B for 3 min and equilibrated for 3 min at 100% A to start the next sample. Total running time was 20 min. Injection volume was 20 µL, and flow rate was 1 mL/min. UV-visible spectra were used to detect flavonoids, and absorbance at 254 nm was used for quantification. 

### 2.11. Water Absorption Index (WAI), Water Solubility Index (WSI) and Expansion Index (EI)

The water absorption index (WAI) and water solubility index (WSI) were assessed before and after the extrusion process [22]. The expansion index (EI) was assessed in seed flour extrudates [22]. 

### 2.12. Statistical Analysis 

Assays were performed in triplicate, and expressed as means ± standard deviation. Data were submitted to analysis of variance (ANOVA) and means were compared by Tukey test (*p* ≤ 0.05). 

## 3. Results and Discussion 

### 3.1. Mesquite Seed Flour Chemical Composition

The chemical composition of mesquite seed flour is shown in Table 1. The main component of mesquite seed flour was nitrogen-free extract (NFE), followed by protein, moisture, crude fiber, fat, and ash. This chemical composition is similar to other seeds of *Prosopis* species, rich in protein and fiber and low in total fat. Previous studies have reported protein contents of 32.3, 62.1 and 30.9 g/100 g in seeds of *P. alba* [9], cotyledons of *P. alba* [14], and seed flour of *P. laevigata* [15], respectively. Previous reported fiber contents in cotyledons of *P. alba* [14], and the seed flour of *P. laevigata* [15] were 9 and 8.3 g/100 g, respectively. Total fat contents of 12.2 and 4 g/100 g have been reported for cotyledons of *P. alba* [14], and seed flour of *P. laevigata* [15], respectively. 

Considering legumes seeds from a different genera, the previously reported contents in white lupine of protein, crude fiber, and fat were 34.6, 12.6, and 10 g/100 g, respectively [23]. These values for lentils and common beans were 26.9 and 19.5, 3.1 and 4.4, and 0.8 and 2.4 g/100 g, respectively [23]. Seed flour of *P. laevigata* maintains the healthy nutritional traits, low fat content, and high content of protein and fiber found in legumes, highlighting the potential industrialization of this underutilized legume seed.

### 3.2. Mesquite Seed Flour Amino Acids Profile

The full amino acid profile of the mesquite seed flour is shown in Table 2. In the present work, four amino acids (Glu, Arg, Asp, and Leu) represented more than 45% of total amino acids, a behavior previously reported for seeds of *P. alba* [14], peas, and common beans [24], but not presented in the soybean [25]. Considering the Food and Agriculture Organization (FAO)-recommended amino acid scoring patterns for humans aged older than 3 years [26], in the present work, Val was the only limiting amino acid in mesquite seed flour. Meanwhile, in seeds of *P. alba*, Lys, Trp, and Thr have been previously reported as limiting amino acids [14]. Trp and sulfur-containing amino acids have been reported as limiting amino acids for peas and common beans, respectively [24]. The seed flour of mesquite is a valuable plant material as a source of good quality protein.

### 3.3. Total Phenolic Compounds, Radical Scavenging Capacity, and Absorbance at 290 nm

Total phenolic compound content, DPPH radical scavenging capacity, and absorbance at 290 nm of seed flour are shown in Table 3. Total phenolic compound content reported here for mesquite seed flour is similar to the previous reported value for *P. laevigata* [15], lower than the value reported for cotyledons of *P. alba* [14], and higher than the respective values reported for lupine, peas, lentils, and common beans [23]. In the present work, the extrusion cooking of mesquite seed flour decreases the content of total phenolic compounds slightly but significantly. DPPH radical scavenging capacity reported here for mesquite seed flour is similar to the previously reported value for *P. laevigata* [15] and higher than the respective values reported for lupine, peas, lentils, and common beans [23]. In the present work, the extrusion cooking of mesquite seed flour increase the DPPH radical scavenging capacity slightly but significantly. The absorbance at 290 nm as an indicator of generation of Maillard reaction products (MRP) was similar in raw and extruded seed flour, suggesting that the extrusion process does not trigger the generation of MRP. 

In previous works the generation of Maillard reaction products (assessed as changes in absorbance at 290 nm) during thermal treatment have been associated with increases in the total phenolic compound content and radical scavenging capacity in pods flours of *P. laevigata* [15], flours of carob pods [27], and in synthetic media [20]. The reaction between reducing sugars and amines during thermal treatments produces low molecular weight heterocycles, which can be detected by increases in UV absorbance at 290 nm [20]. These Maillard reaction products have an important free radical scavenging capacity, with reducing activity over the Folin–Ciocalteu reagent in the quantification of total phenolic compounds [15,20]. In some cases, MRP may generate negative effects on human health, resulting in anti-nutritional properties such as the loss of essential amino acids [28]. Flours of mesquite pods have been described as a material very prone to Maillard reaction products generation during baking process [15]. In the presented work the extrusion cooking conditions used for mesquite seed flour processing involves high temperature (150 °C), short time periods (seconds), and low water content (16%); these conditions have been previously suggested for control of MRP generation [28]. Mesquite seed flour is an important source of phenolic compounds with high radical scavenging capacity compared with other legume seeds. Mesquite seed flour extrusion cooking does not affect phenolic compound content or radical scavenging capacity, and does not promote the generation of Maillard reaction products, supporting the use of extrusion technology as an excellent option for mesquite seed flour processing and industrialization. 

### 3.4. Flavonoids

The content of apigenin in raw and extruded mesquite seed flour is shown in Table 3. In the present work, mesquite seed flour was investigated for the presence of myricetin, quercetin, kaempferol, apigenin, daidzein, and genistein. From these flavonoids only the presence of apigenin was confirmed by retention time and UV-VIS spectra. Given the used methodology, the apigenin found in mesquite seed flours corresponds to O-glycosides of apigenin, which were stable to the extrusion cooking process. Previous works reported the presence of C-glycosides of apigenin in pods flours of *P. alba* [11,13,14], and *Prosopis nigra* [29] without quantification. Quercetin, myricetin, and luteolin glycosides have been found in pods flours of *P. alba* [13], Legume seeds have been described as an excellent source of flavonoids; soybean-daidzein and genistein; pea-quercetin, apigenin and kaempferol; common bean-quercetin and kaempferol [21,30,31]. Dietary flavonoids from legumes, including apigenin, have been related with healthy effects on human metabolism through modulation of oxidative stress, hormone function, energetic metabolism, gene expression, and epigenetic process [32,33,34,35]. Mesquite seed flour is a source of apigenin, an important active component with healthy implications for humans.

### 3.5. Water Absorption Index (WAI), Water Solubility Index (WSI) and Expansion Index (EI)

Water absorption index (WAI) and water solubility index (WSI) of raw and extruded mesquite seed flour, together with the expansion index of extruded mesquite seed flour, is shown in Table 3. No previous works were found with respect to *Prosopis* seed flour extrusion. In the present work the extrusion cooking process of mesquite seed flour increases the WAI significantly. Water absorption indicates the amount of water immobilized by the material, as the dispersion by denaturalization of macromolecules such as proteins and starch during extrusion increase the WAI [36], an effect previously described in common bean flours [37]. In the present work the extrusion cooking process of mesquite seed flour decreases the WSI significantly. Water solubility indicates the amount of small molecules solubilized in water, which can be components of the extruded material or can be generated by molecular damage during the extrusion process [36]. Raw mesquite seed flour is a material with water-soluble molecules trapped in the structure formed by the macromolecules denaturalized during extrusion, decreasing the WSI. In the present work the extrusion cooking process of mesquite seed flour produces an extruded material with a poor expansion index, which can be due to the high protein and fiber content in the raw material. High contents of protein and fiber decreased the expansion index of extrudates [38], an effect previously described in corn and common bean-extruded flour blends [39]. Another reported effect of legume seed flour extrusion is the improvement of protein digestibility [40]. The extrusion cooking process of mesquite seed flour modifies the molecular structure of components, improving the nutritional value of the material. Future studies must be conducted in order to develop new extruded products based on mesquite seed flour or in blends with cereals. 

## 4. Conclusions

Mesquite seed flour is a valuable plant food rich in good quality protein and active compounds. The extrusion cooking process of mesquite seed flour is an optional and versatile technology useful in the development of functional foods and industrialization of this underutilized legume.

## Figures and Tables

**Table 1:** Chemical composition of mesquite (*Prosopis laevigata*) seed flour.

Component	g/100 g *
Moisture	8.28 ± 0.15
Ash	4.14 ± 0.03
Protein	36.51 ± 0.36
Fat	4.83 ± 0.04
Crude Fiber	7.73 ± 0.46
NFE	38.45 ± 0.66

NFE: nitrogen-free extract. * Values expressed as means ± standard deviation.

**Table 2:** Amino acid (AA) profile of mesquite seed flour (mg/g protein).

AA	Seed Flour	* FAO, 2013
Asp	83.4 ± 1.27	
Thr	29.8 ± 0.35	25
Ser	48.1 ± 0.15	
Glu	177.2 ± 2.08	
Pro	62.6 ± 0.95	
Gly	50.6 ± 0.05	
Ala	43.1 ± 0.29	
Val	34.8 ± 0.31	40
Ile	29.2 ± 0.1	30
Leu	69.1 ± 0.45	61
Tyr	22.8 ± 0.61	
Phe	35.6 ± 0.49	
His	24.2 ± 0.3	16
Lys	54.8 ± 0.41	48
Arg	112.2 ± 1.93	
Cis	25.9 ± 0.12	
Met	9.1 ± 0.21	
Trp	6.5 ± 0.22	6.6
Met + Cis	34.9 ± 0.34	23
Phe + Tyr	58.4 ± 1.10	41

* Food and Agriculture Organization (FAO)-recommended amino-acid scoring patterns for humans aged older than 3 years [24]. Values expressed as means ± standard deviation.

**Table 3:** Properties of raw and extruded mesquite seed flour.

	Seed Flour *	Extruded Seed Flour *
Total phenolics (mg GAE/g)	6.68 ± 0.05 ^a^	6.46 ± 0.06 ^b^
DPPH (mg AAE/g)	9.11 ± 0.11 ^a^	9.32 ± 0.12 ^b^
Abs 290 nm	0.13 ± 0.01 ^a^	0.12 ± 0.01 ^a^
Apigenin (mg/kg)	41.6 ± 0.51 ^a^	39.52 ± 0.47 ^b^
WAI	2.53 ± 0.01 ^a^	3.47 ± 0.11 ^b^
WSI (%)	36.36 ± 0.57 ^a^	30.52 ± 0.99 ^b^
Expansion index		1

* Values were expressed as means ± standard deviation (*n* = 3). Means accompanied by the same letter in the same line indicate no significant difference between samples (*p* < 0.05). WAI—water absorption index; WSI—water solubility index; GAE—gallic acid equivalent; AAE—ascorbic acid equivalent; DPPH—2-2-diphenyl-1-picrylhydrazyl.
